# Supplementary material for: The interactome of intact mitochondria by cross-linking mass spectrometry provides evidence for coexisting respiratory supercomplexes
Source: Mol Cell Proteomics. 2017 Dec 8;17(2):216–32. doi: 10.1074/mcp.RA117.000470 (PMC5795388; doi:10.1074/mcp.RA117.000470)
Supplement: Supplemental Data [file supp_RA117.000470_133922_0_supp_23978_fzffwf.zip › spectra_annotation/mito_spectra_annotation/303-1-11-1-2-1.pdf]

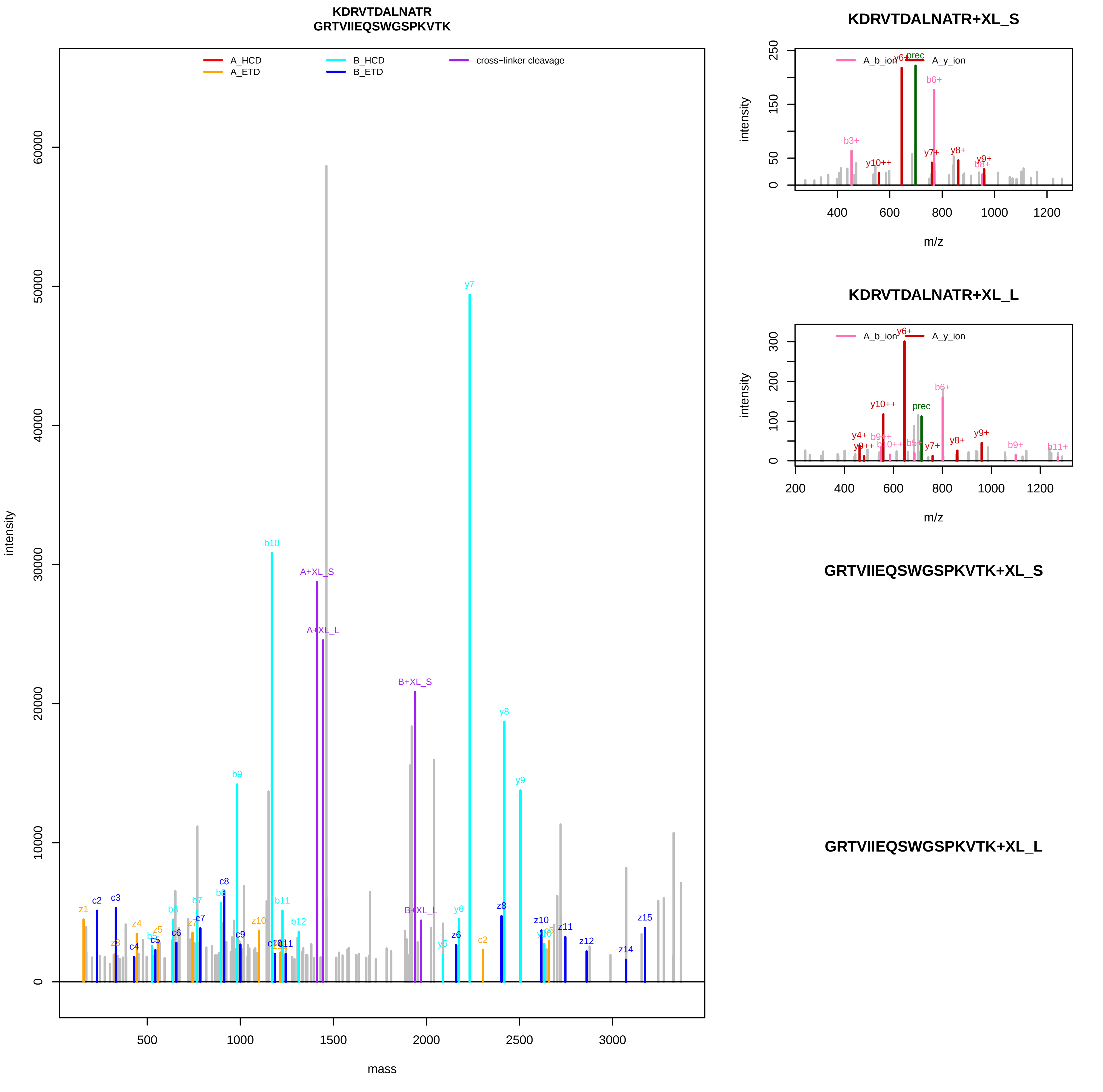
KDRVTDALNATR
GRTVIIEQSWGSPKVTK
A_HCD
A_ETD
B_HCD
B_ETD
cross−linker cleavage
intensity
mass
y7
b10
A+XL_S
A+XL_L
B+XL_S
B+XL_L
y8
y9
b9
c8
c2
c3
z1
z4
z5
c5
c6
b6
b7
c7
c9
z10
b11
b12
y6
z6
y5
c2
z8
z10
c5
z11
z12
z14
z15
0
10000
20000
30000
40000
50000
60000
500
1000
1500
2000
2500
3000
KDRVTDALNATR+XL_S
A_b_ion
A_y_ion
y6+
prec
b6+
b3+
y10++
y7+
y8+
y9+
intensity
m/z
0
50
150
250
400
600
800
1000
1200
KDRVTDALNATR+XL_L
A_b_ion
A_y_ion
y6+
b6+
y10++
prec
y4+
y8+
y9+
y7+
b9+
b11+
intensity
m/z
0
100
200
300
200
400
600
800
1000
1200
GRTVIIEQSWGSPKVTK+XL_S
GRTVIIEQSWGSPKVTK+XL_L
